# Supplementary material for: Covalently Linked Peptides and Membrane Potential Enable CyaA Segment Translocation
Source: Adv Sci (Weinh). 2026 Aug 21:e77133. Online ahead of print. doi: 10.1002/advs.77133 (PMC13495951; doi:10.1002/advs.77133)
Supplement: Supplementary file 1 [file ADVS-9999-e77133-s001.pdf]

Supporting Information

Covalently linked peptides and membrane potential enable CyaA segment translocation

Gaia Scilironi, Nicolas Carvalho, Jacinthe Frangieh, Corentin Léger, Dorothée Raoux-Barbot, J. Iñaki Guijarro, Daniel Ladant, Sophie Cribier, Nicolas Rodriguez, Alexandre Chenal

**Condition A**		**Condition B**		**Statistical significance (*p* value)**	
**Molecule**	**Membrane potential (mV)**	**Molecule**	**Membrane potential (mV)**	**Fisher's exact test**	**Mann-Whitney U test**
FITC-dextran	-150	FITC-dextran	0	1.00	0.31
TAMRA-P454-mut2	-150	TAMRA-P454-mut2	0	0.33	0.45
TAMRA-P454	0	FITC-dextran	0	0.03	0.02
TAMRA-P454	-150	FITC-dextran	-150	0.05	0.05
TAMRA-P454-mut2	0	FITC-dextran	0	1.00	0.82
TAMRA-P454-mut2	-150	FITC-dextran	-150	0.50	0.50
TAMRA-P454	0	TAMRA-P454-mut2	0	0.01	0.01
TAMRA-P454	-150	TAMRA-P454-mut2	-150	0.05	0.05
TAMRA-P233	0	FITC-dextran	0	1.00	0.17
TAMRA-P233	-80	FITC-dextran	-150	0.05	0.05
TAMRA-P233	-150	FITC-dextran	-150	0.05	0.05
TAMRA-P233 (in the presence of unlabeled P454)	0	FITC-dextran	0	1.00	0.11

TAMRA-P233-P454	0	FITC-dextran	0	0.01	0.01

Table S1: Statistical significance of peptides and FITC-dextran membrane translocation assesses as described in the **Experimental Section**, using the Fisher exact test and the Mann-Whitney U test. Statistical significance of peptide/FITC-dextran membrane translocation is assessed evaluating translocation in condition A with respect to translocation in condition B. Results were considered statistically significant for p value ≤ 0.05.

	**K_d (µM)**	**K_d error (µM)**	**K_x**	**ΔG_{Kx} (kcal/mol)**	**K_d (µM)**	**K_d error (µM)**	**K_x**	**ΔG_{Kx} (kcal/mol)**
**Method**	Tryptophan fluorescence	Tryptophan fluorescence	Tryptophan fluorescence	Tryptophan fluorescence	B2LiVe	B2LiVe	B2LiVe	B2LiVe
**DOPC: POPG 8:2**	582	46	$9.53^{E}4$	- 6.8	255	70	$2.18^{E}5$	- 7.3
**POPC: POPG 8:2**	1171	47	$4.74^{E}4$	- 6.4	462	94	$1.20^{E}5$	- 6.9
**POPC**	3095	378	$1.79^{E}4$	- 5.8	1127	804	$4.92^{E}4$	- 6.4

Table S2: Thermodynamic parameters of the solution to membrane partitioning of 5 µM of P233 obtained by tryptophan fluorescence and B2LiVe methods, at 25°C in the presence of increasing (0-10 mM) concentrations of lipids, using SUVs composed of DOPC:POPG at a molar ratio of 8:2, POPC:POPG at a molar ratio of 8:2, and POPC 100%.

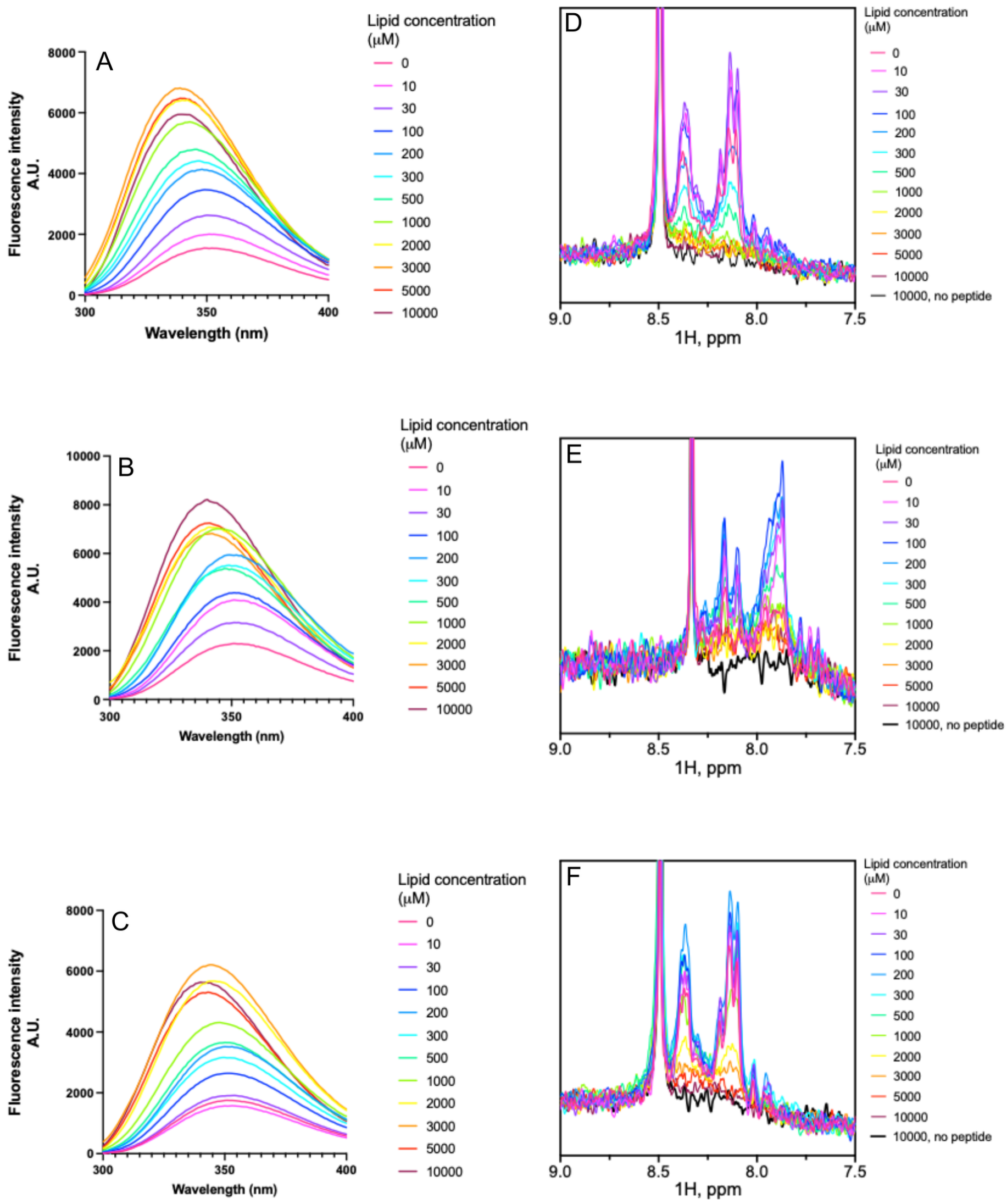

Figure S1: Solution to membrane partitioning of 5 µM of P233 monitored by changes of the tryptophan fluorescence (**A, B, C**), and by the B2LiVe method (**D, E, F**) at 25°C. The concentration of lipids ranged from 0 (in fuchsia) to 10 mM (in burgundy). Three different SUVs compositions were tested: A) and D): DOPC:POPG 8:2, B) and E): POPC:POPG 8:2 and C) and F): POPC. In A, B, C: membrane partitioning is indicated by a shift towards lower wavelength values of the maximum fluorescence emission wavelength and by an increase of the fluorescence intensity. In D, E, F: membrane partitioning is followed by disappearance of the amide resonance signal. The sharp peak around 8.5 ppm is due to a trace impurity of formic acid contained in D_2O. The NMR spectrum of SUVs in the absence of peptide is reported as a black line.

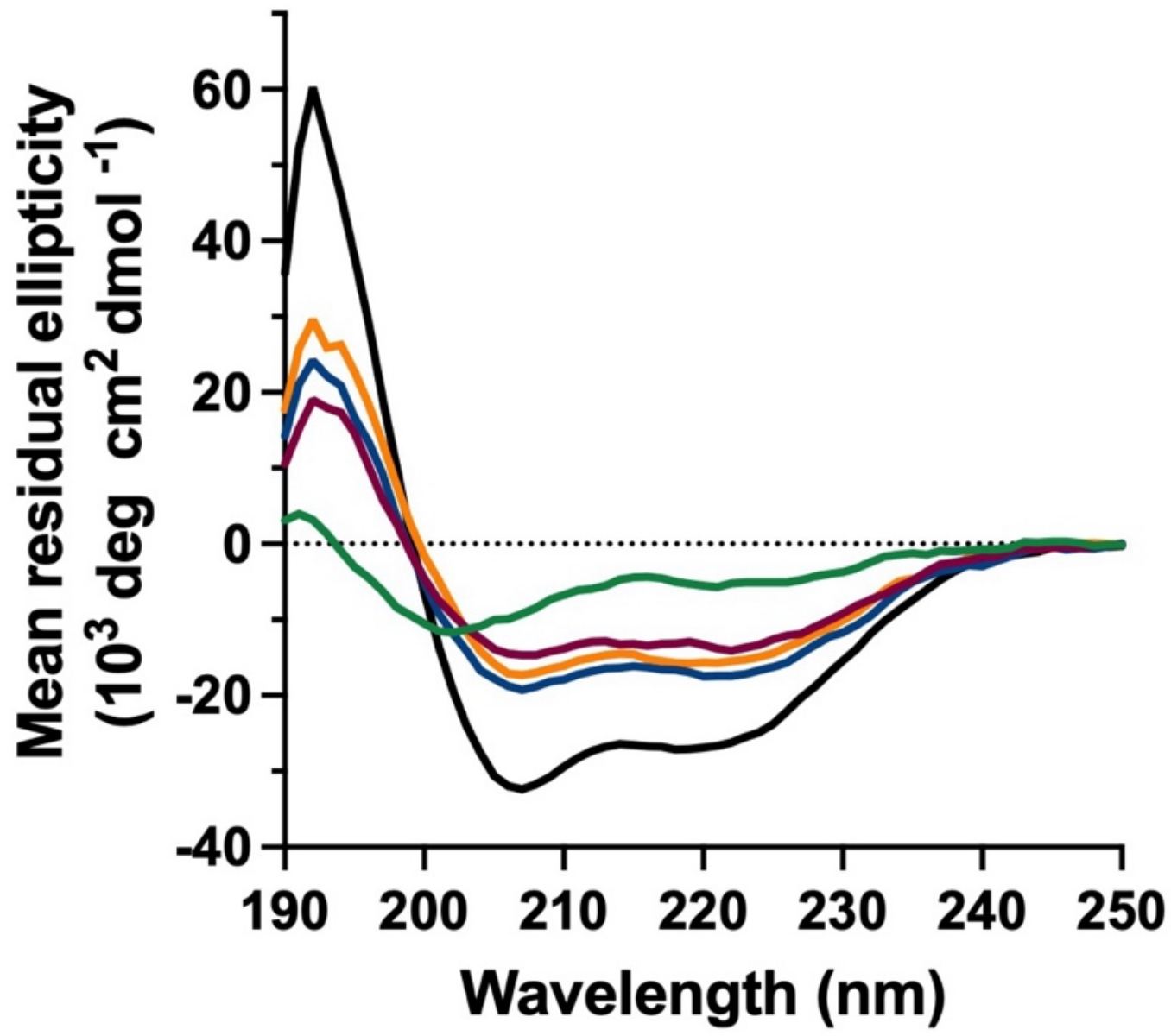

Figure S2: Far-UV Synchrotron radiation circular dichroism (SRCD) spectra of 100 µM P454 and 100 µM P233 recorded in the absence (green line) and in the presence of SUVs (6 mM of lipids) composed of POPC (burgundy line), POPC:POPG at a molar ratio of 8:2 (blue line), or DOPC:POPG at a molar ratio of 8:2 (orange line), as well as in the presence of 40% TFE (black line) in buffer A at 25 °C.

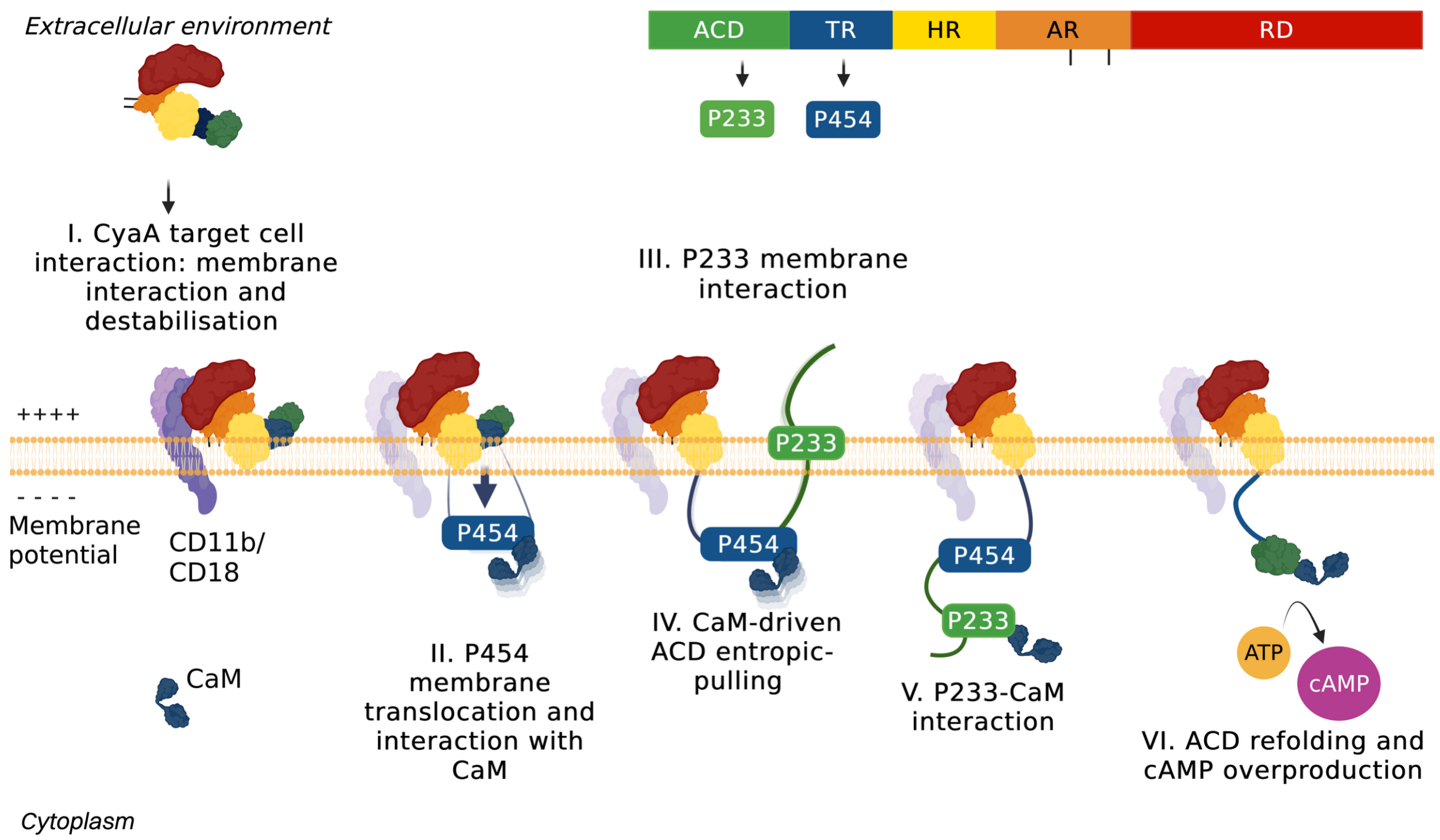

Figure S3: Schematic representation of the proposed mechanism of CyaA membrane translocation. CyaA interacts with the CD11b/CD18 cell receptor exposed on the surface of mature myeloid phagocytes. Alternatively, CyaA may directly interact with the plasma membrane of eukaryotic cells. In both cases, CyaA inserts its acyl chains and its hydrophobic region (HR, containing several hydrophobic segments) into the membrane (I). The P454 region interacts with the plasma membrane and translocates across the lipid bilayer. Once in the *trans* compartment of the membrane, P454 binds to CaM, resulting in entropic pulling of TR inside the cell cytoplasm (II). The P233 segment, upon membrane interaction docks its flanking regions from ACD to the membrane (III). P454 translocation may facilitate the translocation of other protein segments: we propose that formation of the P454:CaM complex generates an entropic pulling force that promotes ACD unfolding and drives its translocation (IV). Supported by membrane potential and by the entropic pulling force produced by the P454:CaM complex, the P233 region translocates and binds to CaM (V). ACD achieves its translocation. ACD folds upon CaM binding and overproduces cAMP from cellular ATP at high turnover rate (VI). CyaA organization is reported on the upper left section of the Figure. CyaA domains are represented as colored rectangles: RD in red, AR in orange, HR in yellow, TR in blue, ACD in green. P454 is represented as a blue rectangle, P233 is represented as a green rectangle. CaM is shown in dark blue. CD11b/CD18 receptor is shown in violet. The membrane is represented in yellow. ATP is represented as a dark yellow circle, cAMP as pink circle. Molecules are not at scale. Made with Biorender.
